# Supplementary material for: Damage-induced IL-18 stimulates thymic NK cells limiting endogenous tissue regeneration
Source: Nat Immunol. 2025 Sep 11;26(10):1699–711. doi: 10.1038/s41590-025-02270-z (PMC12479347; doi:10.1038/s41590-025-02270-z)
Supplement: Supplementary file 1 — Reporting Summary [file 41590_2025_2270_MOESM1_ESM.pdf]

nature portfolio

Corresponding author(s): Jarrod Dudakov, Marcel van den Brink

Last updated by author(s): 2/15/2025

Reporting Summary

Nature Portfolio wishes to improve the reproducibility of the work that we publish. This form provides structure for consistency and transparency in reporting. For further information on Nature Portfolio policies, see our Editorial Policies and the Editorial Policy Checklist.

Statistics

For all statistical analyses, confirm that the following items are present in the figure legend, table legend, main text, or Methods section.

n/a	Confirmed	
☐	☒	The exact sample size (*n*) for each experimental group/condition, given as a discrete number and unit of measurement
☐	☒	A statement on whether measurements were taken from distinct samples or whether the same sample was measured repeatedly
☐	☒	The statistical test(s) used AND whether they are one- or two-sided<br>*Only common tests should be described solely by name; describe more complex techniques in the Methods section.*
☐	☒	A description of all covariates tested
☐	☒	A description of any assumptions or corrections, such as tests of normality and adjustment for multiple comparisons
☐	☒	A full description of the statistical parameters including central tendency (e.g. means) or other basic estimates (e.g. regression coefficient) AND variation (e.g. standard deviation) or associated estimates of uncertainty (e.g. confidence intervals)
☒	☐	For null hypothesis testing, the test statistic (e.g. *F*, *t*, *r*) with confidence intervals, effect sizes, degrees of freedom and *P* value noted<br>*Give P values as exact values whenever suitable.*
☒	☐	For Bayesian analysis, information on the choice of priors and Markov chain Monte Carlo settings
☒	☐	For hierarchical and complex designs, identification of the appropriate level for tests and full reporting of outcomes
☒	☐	Estimates of effect sizes (e.g. Cohen's *d*, Pearson's *r*), indicating how they were calculated

Our web collection on statistics for biologists contains articles on many of the points above.

Software and code

Policy information about availability of computer code

Data collection

Cells were sorted on an Aria II or Symphony S6 (BD Biosciences) using FACSDiva (BD Biosciences, Franklin Lakes, NJ). Flow cytometry analysis was performed on a Symphony A6 using FACSDiva Software (BD Biosciences). Sequencing data were collected on Illumina Nextseq or Novaseq platforms. Tissue sections were imaged on a Zeiss LSM 880 confocal microscope. Formalin-fixed paraffin-embedded thymus tissue section images were acquired on the Vectra Polaris Quantitative Pathology Imaging System (Akoya Biosciences, Marlborough, MA).

Data analysis

#1. Flow cytometry data were analyzed using FlowJo (v10.7.1, TreeStar Software) and GraphPad Prism (v10; GraphPad Software, LLC). Data was collected and cells sorted on BD cytometers using FACSDiva software.

#2. All single cell RNA-seq data were analyzed using Cell Ranger (v7.0.1; 10x Genomics). The shunPykeR adapted Jupyter notebooks, R notebooks, and the assorted conda (.yml) and renv (renv.lock) environment files to reproduce analyses and figure creation for this manuscript can be found at https://github.com/kousaa/Kousa-et-al-2024-NI. The app.R code that launches the ThymoSight app, together with the python notebooks used to create consistent annotation fields, reanalyze and integrate the public datasets with ours have been submitted on GitHub at https://github.com/FredHutch/thymosight. The server hosting the interactive app can be accessed at www.thymosight.org. Environments and packages can be found listed below.

#3. Other software: Cytoscape (v3.10.0); HALO image analysis software (v4.0)

Environment names and requirements for scRNAseq analsysis:

name: Kousa-et-al-2024-NI (main), channels: conda-forge, defaults, dependencies: -alabaster=0.7.12, -anndata=0.8.0, -appnope=0.1.2, -argon2-cffi=21.3.0, -argon2-cffi-bindings=21.2.0, -arpack=3.7.0, -asttokens=2.0.5, -attrs=21.4.0, -babel=2.10.3, -backcall=0.2.0, -

beautifulsoup4=4.11.1, -bleach=4.1.0, -brotli=1.0.9, -brotli-bin=1.0.9, -brotlipy=0.7.0, -ca-certificates=2022.6.15.1, -certifi=2022.6.15.1, -
cffi=1.15.1, -charset-normalizer=2.1.1, -colorama=0.4.5, -cryptography=37.0.4, -cycler=0.11.0, -debugpy=1.5.1, -decorator=5.1.1, -
defusedxml=0.7.1, -docutils=0.19, -entrypoints=0.4, -executing=0.8.3, -fonttools=4.37.1, -freetype=2.10.4, -glpk=4.65, -gmp=6.2.1, -
h5py=3.7.0, -hdf5=1.10.6, -icu=58.2, -igraph=0.9.10, -imagesize=1.4.1, -importlib-metadata=4.11.4, -importlib_metadata=4.11.4, -
importlib_resources=5.2.0, -ipykernel=6.15.2, -ipython=8.4.0, -ipython_genutils=0.2.0, -jedi=0.18.1, -jinja2=3.0.3, -joblib=1.1.0, -jpeg=9e, -
jsonschema=4.4.0, -jupyter_client=7.3.5, -jupyter_core=4.10.0, -jupyterlab_pygments=0.1.2, -kiwisolver=1.4.4, -lcms2=2.12, -
leidenalg=0.8.10, -libblas=3.9.0, -libbrotlicommon=1.0.9, -libbrotlidec=1.0.9, -libbrotlienc=1.0.9, -libcblas=3.9.0, -libcxx=14.0.6, -libffi=3.3, -
libgfortran=4.0.0, -libgfortran4=7.5.0, -libiconv=1.17, -liblapack=3.9.0, -libllvm11=11.1.0, -libopenblas=0.3.12, -libpng=1.6.37, -
libsodium=1.0.18, -libtiff=4.2.0, -libwebp-base=1.2.4, -libxml2=2.9.14, -llvm-openmp=14.0.4, -llvmlite=0.38.0, -lz4-c=1.9.3, -markupsafe=2.1.1,
-matplotlib-base=3.5.3, -matplotlib-inline=0.1.6, -metis=5.1.0, -mistune=0.8.4, -mpfr=4.1.0, -munkres=1.1.4, -natsort=8.2.0, -nbclient=0.5.13, -
nbconvert=6.4.4, -nbformat=5.3.0, -ncurses=6.3, -nest-asyncio=1.5.5, -networkx=2.8.6, -notebook=6.4.12, -numba=0.55.1, -olefile=0.46, -
openssl=1.1.1q, -packaging=21.3, -pandas=1.4.4, -pandocfilters=1.5.0, -parso=0.8.3, -patsy=0.5.2, -pexpect=4.8.0, -pickleshare=0.7.5, -
pip=22.1.2, -prometheus_client=0.14.1, -prompt-toolkit=3.0.20, -psutil=5.9.0, -ptyprocess=0.7.0, -pure_eval=0.2.2, -pycparser=2.21, -
pygments=2.11.2, -pynndescent=0.5.7, -pyopenssl=22.0.0, -pyparsing=3.0.9, -pyrsistent=0.18.0, -pysocks=1.7.1, -python=3.8.13, -python-
dateutil=2.8.2, -python-fastjsonschema=2.16.2, -python-igraph=0.9.11, -python_abi=3.8, -pytz=2022.2.1, -pyzmq=23.2.0, -readline=8.1.2, -
scanpy=1.9.1, -scikit-learn=1.1.2, -scipy=1.5.3, -seaborn=0.12.0, -seaborn-base=0.12.0, -send2trash=1.8.0, -session-info=1.0.0, -
setuptools=63.4.1, -six=1.16.0, -snowballstemmer=2.2.0, -soupsieve=2.3.1, -sphinx=5.1.1, -sphinxcontrib-applehelp=1.0.2, -sphinxcontrib-
devhelp=1.0.2, -sphinxcontrib-htmlhelp=2.0.0, -sphinxcontrib-jsmath=1.0.1, -sphinxcontrib-qthelp=1.0.3, -sphinxcontrib-serializinghtml=1.1.5,
-sqlite=3.39.2, -stack_data=0.2.0, -statsmodels=0.13.2, -stdlib-list=0.7.0, -suitesparse=5.10.1, -tbb=2021.5.0, -terminado=0.13.1, -
testpath=0.6.0, -texttable=1.6.4, -threadpoolctl=3.1.0, -tk=8.6.12, -tornado=6.2, -tqdm=4.64.1, -traitlets=5.1.1, -typing-extensions=4.3.0, -
typing_extensions=4.3.0, -umap-learn=0.5.3, -unicodedata2=14.0.0, -urllib3=1.26.11, -wcwidth=0.2.5, -webencodings=0.5.1, -wheel=0.37.1, -
xz=5.2.5, -zeromq=4.3.4, -zipp=3.8.0, -zlib=1.2.12, -zstd=1.4.9, -pip:, -aniso8601==9.0.1, -anndata2ri==1.1, -annoy==1.17.1, -backports-
zoneinfo==0.2.1, -biomart==0.9.2, -chardet==4.0.0, -click==8.1.3, -cmake==3.24.1.1, -cython==0.29.32, -dunamai==1.13.0, -et-xmlfile==1.1.0,
-fa2==0.3.5, -fbpca==1.0, -fcsparser==0.2.4, -feather-format==0.4.1, -flask==1.1.2, -flask-cors==3.0.10, -flask-restful==0.3.9, -geosketch==1.2,
-get-version==3.5.4, -harmonypy==0.0.6, -harmonyts==0.1.4, -idna==2.10, -imageio==2.22.1, -intervaltree==3.1.0, -itsdangerous==2.1.2, -
loompy==3.0.7, -mousipy==0.0.5, -nbstripout==0.6.1, -numexpr==2.8.3, -numpy==1.20.2, -numpy-groupies==0.9.19, -openpyxl==3.0.10, -
palantir==1.0.1, -phenograph==1.5.7, -pillow==9.2.0, -pyarrow==9.0.0, -pyreadr==0.4.6, -python-slugify==5.0.2, -pytz-deprecation-
shim==0.1.0.post0, -pywavelets==1.3.0, -requests==2.25.1, -rpy2==3.5.4, -scanorama==1.7.2, -scikit-image==0.19.3, -scrublet==0.2.3, -
scvelo==0.2.4, -sklearn==0.0, -sortedcontainers==2.4.0, -tables==3.7.0, -text-unidecode==1.3, -tifffile==2022.8.12, -tzdata==2022.2, -
tzlocal==4.2, -watermark==2.3.1, -werkzeug==2.2.2, -wrapt==1.15.0, -xlrd==2.0.1, -xlsxwriter==3.0.3

name: Kousa-et-al-2024-NI (dynamo), channels: defaults, dependencies: -anyio=4.2.0, -appnope=0.1.2, -argon2-cffi=21.3.0, -argon2-cffi-
bindings=21.2.0, -asttokens=2.0.5, -async-lru=2.0.4, -babel=2.11.0, -beautifulsoup4=4.12.2, -bleach=4.1.0, -brotli-python=1.0.9, -bzip2=1.0.8, -
ca-certificates=2023.12.12, -certifi=2024.2.2, -cffi=1.16.0, -charset-normalizer=2.0.4, -comm=0.1.2, -debugpy=1.6.7, -decorator=5.1.1, -
defusedxml=0.7.1, -executing=0.8.3, -ipykernel=6.28.0, -ipython=8.20.0, -jedi=0.18.1, -json5=0.9.6, -jsonschema=4.19.2, -jsonschema-
specifications=2023.7.1, -jupyter-lsp=2.2.0, -jupyter_client=8.6.0, -jupyter_core=5.5.0, -jupyter_events=0.8.0, -jupyter_server=2.10.0, -
jupyter_server_terminals=0.4.4, -jupyterlab=4.0.11, -jupyterlab_pygments=0.1.2, -jupyterlab_server=2.25.1, -libcxx=14.0.6, -libffi=3.4.4, -
libsodium=1.0.18, -matplotlib-inline=0.1.6, -mistune=2.0.4, -nbclient=0.8.0, -nbconvert=7.10.0, -nbformat=5.9.2, -ncurses=6.4, -nest-
asyncio=1.6.0, -notebook=7.0.8, -notebook-shim=0.2.3, -openssl=3.0.13, -overrides=7.4.0, -pandocfilters=1.5.0, -parso=0.8.3, -pexpect=4.8.0,
-pip=23.3.1, -prometheus_client=0.14.1, -prompt-toolkit=3.0.43, -prompt_toolkit=3.0.43, -psutil=5.9.0, -ptyprocess=0.7.0, -pure_eval=0.2.2, -
pycparser=2.21, -pysocks=1.7.1, -python=3.11.8, -python-fastjsonschema=2.16.2, -python-json-logger=2.0.7, -pyyaml=6.0.1, -pyzmq=25.1.2, -
readline=8.2, -referencing=0.30.2, -requests=2.31.0, -rfc3339-validator=0.1.4, -rfc3986-validator=0.1.1, -rpds-py=0.10.6, -send2trash=1.8.2, -
setuptools=68.2.2, -six=1.16.0, -sniffio=1.3.0, -soupsieve=2.5, -sqlite=3.41.2, -stack_data=0.2.0, -terminado=0.17.1, -tinycss2=1.2.1, -
tk=8.6.12, -tornado=6.3.3, -traitlets=5.7.1, -typing_extensions=4.9.0, -urllib3=2.1.0, -wcwidth=0.2.5, -webencodings=0.5.1, -websocket-
client=0.58.0, -wheel=0.41.2, -xz=5.4.6, -yaml=0.2.5, -zeromq=4.3.5, -zlib=1.2.13, -pip:, -absl-py==2.1.0, -aiohttp==3.9.3, -aiosignal==1.3.1, -
anndata==0.10.5.post1, -array-api-compat==1.4.1, -attrs==23.2.0, -cellrank==2.0.3.dev5+g721c59f, -cfgv==3.4.0, -chex==0.1.85, -click==8.1.7,
-colorcet==3.1.0, -contextlib2==21.6.0, -contourpy==1.2.0, -cycler==0.12.1, -distlib==0.3.8, -docrep==0.3.2, -dunamai==1.19.2, -dynamo-
release==1.4.0, -et-xmlfile==1.1.0, -etils==1.7.0, -filelock==3.13.1, -flax==0.8.1, -fonttools==4.49.0, -frozenlist==1.4.1, -fsspec==2024.2.0, -get-
version==3.5.5, -h5py==3.10.0, -identify==2.5.35, -idna==3.6, -igraph==0.10.8, -importlib-resources==6.1.2, -jax==0.4.25, -jaxlib==0.4.25, -
jinja2==3.0.3, -joblib==1.3.2, -kiwisolver==1.4.5, -lightning==2.1.4, -lightning-utilities==0.10.1, -llvmlite==0.42.0, -loompy==3.0.7, -
louvain==0.8.0, -markdown-it-py==3.0.0, -markupsafe==2.1.5, -matplotlib==3.6.3, -mdurl==0.1.2, -ml-collections==0.1.1, -ml-dtypes==0.3.2, -
mpmath==1.3.0, -msgpack==1.0.8, -mudata==0.2.3, -multidict==6.0.5, -multipledispatch==1.0.0, -natsort==8.4.0, -networkx==3.2.1, -
nodeenv==1.8.0, -numba==0.59.0, -numdifftools==0.9.41, -numpy==1.26.4, -numpy-groupies==0.10.2, -numpyro==0.13.2, -openpyxl==3.1.2,
-opt-einsum==3.3.0, -optax==0.1.9, -orbax-checkpoint==0.5.3, -packaging==23.2, -pandas==2.2.1, -patsy==0.5.6, -pillow==10.2.0, -
platformdirs==4.2.0, -pre-commit==3.6.2, -progressbar2==4.4.1, -protobuf==4.25.3, -pygam==0.9.1, -pygments==2.17.2, -pygpcca==1.0.4, -
pynndescent==0.5.11, -pyparsing==3.1.1, -pyro-api==0.1.2, -pyro-ppl==1.9.0, -python-dateutil==2.9.0, -python-utils==3.8.2, -pytorch-
lightning==2.2.0.post0, -pytz==2024.1, -rich==13.7.1, -scanpy==1.9.8, -scikit-learn==1.1.3, -scipy==1.11.4, -scvelo==0.3.1, -scvi-tools==1.1.1, -
seaborn==0.13.2, -session-info==1.0.0, -statsmodels==0.14.1, -stdlib-list==0.10.0, -sympy==1.12, -tensorstore==0.1.54, -texttable==1.7.0, -
threadpoolctl==3.3.0, -toolz==0.12.1, -torch==2.2.1, -torchmetrics==1.3.1, -tqdm==4.66.2, -typing-extensions==4.10.0, -tzdata==2024.1, -
umap-learn==0.5.5, -virtualenv==20.25.1, -wrapt==1.16.0, -yarl==1.9.4, -zipp==3.17.0

name: Kousa-et-al-2024-NI (thymosight), channels: -conda-forge, -defaults, dependencies: -anndata=0.10.5.post1, -anyio=4.2.0, -
appnope=0.1.2, -argon2-cffi=21.3.0, -argon2-cffi-bindings=21.2.0, -arpack=3.8.0, -array-api-compat=1.4.1, -asttokens=2.0.5, -async-lru=2.0.4,
-attrs=23.1.0, -babel=2.11.0, -beautifulsoup4=4.12.2, -bleach=4.1.0, -brotli=1.1.0, -brotli-bin=1.1.0, -brotli-python=1.0.9, -bzip2=1.0.8, -c-
ares=1.27.0, -ca-certificates=2024.2.2, -cached-property=1.5.2, -cached_property=1.5.2, -certifi=2024.2.2, -cffi=1.16.0, -charset-
normalizer=2.0.4, -colorama=0.4.6, -contourpy=1.2.0, -cycler=0.12.1, -debugpy=1.6.7, -decorator=5.1.1, -defusedxml=0.7.1, -
exceptiongroup=1.2.0, -executing=0.8.3, -fonttools=4.25.0, -freetype=2.12.1, -get-annotations=0.1.2, -glpk=5.0, -gmp=6.3.0, -h5py=3.10.0, -
hdf5=1.14.3, -icu=73.2, -idna=3.4, -igraph=0.10.10, -ipykernel=6.28.0, -ipython=8.20.0, -jedi=0.18.1, -jinja2=3.1.3, -joblib=1.3.2, -json5=0.9.6, -
jsonschema=4.19.2, -jsonschema-specifications=2023.7.1, -jupyter-lsp=2.2.0, -jupyter_client=8.6.0, -jupyter_core=5.5.0, -
jupyter_events=0.8.0, -jupyter_server=2.10.0, -jupyter_server_terminals=0.4.4, -jupyterlab=4.0.11, -jupyterlab_pygments=0.1.2, -
jupyterlab_server=2.25.1, -kiwisolver=1.4.5, -krb5=1.21.2, -lcms2=2.16, -leidenalg=0.10.2, -lerc=4.0.0, -libaec=1.1.2, -libblas=3.9.0, -
libbrotlicommon=1.1.0, -libbrotlidec=1.1.0, -libbrotlienc=1.1.0, -libcblas=3.9.0, -libcurl=8.5.0, -libcxx=16.0.6, -libdeflate=1.19, -
libedit=3.1.20191231, -libev=4.33, -libexpat=2.5.0, -libffi=3.4.4, -libgfortran=5.0.0, -libgfortran5=13.2.0, -libhwloc=2.9.3, -libiconv=1.17, -
libjpeg-turbo=3.0.0, -liblapack=3.9.0, -libleidenalg=0.11.1, -libllvm14=14.0.6, -libnghttp2=1.58.0, -libopenblas=0.3.26, -libpng=1.6.43, -
libsodium=1.0.18, -libsqlite=3.45.1, -libssh2=1.11.0, -libtiff=4.6.0, -libwebp-base=1.3.2, -libxcb=1.15, -libxml2=2.12.5, -libzlib=1.2.13, -llvm-
openmp=17.0.6, -llvmlite=0.42.0, -markupsafe=2.1.3, -matplotlib-base=3.8.3, -matplotlib-inline=0.1.6, -mistune=2.0.4, -munkres=1.1.4, -

natsort=8.4.0, -nbclient=0.8.0, -nbconvert=7.10.0, -nbformat=5.9.2, -ncurses=6.4, -nest-asyncio=1.6.0, -networkx=3.2.1, -notebook=7.0.8, -notebook-shim=0.2.3, -numba=0.59.0, -numpy=1.26.4, -openjpeg=2.5.0, -openssl=3.2.1, -overrides=7.4.0, -packaging=23.1, -pandas=2.2.1, -pandocfilters=1.5.0, -parso=0.8.3, -patsy=0.5.6, -pexpect=4.8.0, -pillow=10.2.0, -pip=23.3.1, -platformdirs=3.10.0, -prometheus_client=0.14.1, -prompt-toolkit=3.0.43, -prompt_toolkit=3.0.43, -psutil=5.9.0, -pthread-stubs=0.4, -ptyprocess=0.7.0, -pure_eval=0.2.2, -pycparser=2.21, -pygments=2.15.1, -pynndescent=0.5.11, -pyparsing=3.1.1, -pysocks=1.7.1, -python=3.11.8, -python-dateutil=2.8.2, -python-fastjsonschema=2.16.2, -python-igraph=0.11.4, -python-json-logger=2.0.7, -python-tzdata=2024.1, -python_abi=3.11, -pytz=2023.3.post1, -pyyaml=6.0.1, -pyzmq=25.1.2, -readline=8.2, -referencing=0.30.2, -requests=2.31.0, -rfc3339-validator=0.1.4, -rfc3986-validator=0.1.1, -rpds-py=0.10.6, -scanpy=1.9.8, -scikit-learn=1.4.1.post1, -scipy=1.12.0, -seaborn=0.13.2, -seaborn-base=0.13.2, -send2trash=1.8.2, -session-info=1.0.0, -setuptools=68.2.2, -six=1.16.0, -sniffio=1.3.0, -soupsieve=2.5, -sqlite=3.41.2, -stack_data=0.2.0, -statsmodels=0.14.1, -stdlib-list=0.10.0, -tbb=2021.11.0, -terminado=0.17.1, -texttable=1.7.0, -threadpoolctl=3.3.0, -tinycss2=1.2.1, -tk=8.6.13, -tornado=6.3.3, -tqdm=4.66.2, -traitlets=5.7.1, -typing-extensions=4.9.0, -typing_extensions=4.9.0, -tzdata=2024a, -umap-learn=0.5.5, -urllib3=2.1.0, -wcwidth=0.2.5, -webencodings=0.5.1, -websocket-client=0.58.0, -wheel=0.41.2, -xorg-libxau=1.0.11, -xorg-libxdmcp=1.1.3, -xz=5.4.5, -yaml=0.2.5, -zeromq=4.3.5, -zlib=1.2.13, -zstd=1.5.5, -pip:, -annoy==1.17.3, -biomart==0.9.2, -comm==0.2.1, -cython==3.0.8, -et-xmlfile==1.1.0, -harmonypy==0.0.6, -imageio==2.34.0, -ipywidgets==8.1.2, -jupyterlab-widgets==3.0.10, -lazy-loader==0.3, -openpyxl==3.1.2, -scikit-image==0.22.0, -scrublet==0.2.3, -tifffile==2024.2.12, -widgetsnbextension==4.0.10

name: Kousa-et-al-NI (cellchat), -R=4.2.2, -Bioconductor=3.15, Python: 3.8.2 (virtualenv), packages: -BBmisc=1.13, -BH=1.78.0-0, -Biobase=2.56.0, -BiocGenerics=0.42.0, -BiocManager=1.30.19, -BiocParallel=1.30.4, -BiocVersion=3.15.2, -CellChat=1.4.0, -ComplexHeatmap=2.12.1, -DBI=1.1.3, -DelayedArray=0.22.0, -DelayedMatrixStats=1.18.2, -DropletUtils=1.16.0, -EnhancedVolcano=1.14.0, -FNN=1.1.3.1, -Formula=1.2-4, -GenomeInfoDb=1.32.4, -GenomeInfoDbData=1.2.8, -GenomicRanges=1.48.0, -GetoptLong=1.0.5, -GlobalOptions=0.1.2, -HDF5Array=1.24.2, -Hmisc=4.7-1, -IRanges=2.30.1, -KernSmooth=2.23-20, -MASS=7.3-58.1, -MAST=1.22.0, -Matrix=1.5-3, -MatrixGenerics=1.8.1, -MatrixModels=0.5-1, -NMF=0.24.0, -R.methodsS3=1.8.2, -R.oo=1.25.0, -R.utils=2.12.2, -R6=2.5.1, -RANN=2.6.1, -RColorBrewer=1.1-3, -RCurl=1.98-1.9, -ROCR=1.0-11, -RSpectra=0.16-1, -Rcpp=1.0.9, -RcppAnnoy=0.0.20, -RcppArmadillo=0.11.4.2.1, -RcppEigen=0.3.3.9.3, -RcppProgress=0.4.2, -RcppTOML=0.1.7, -Rhdf5lib=1.18.2, -Rtsne=0.16, -S4Vectors=0.34.0, -Seurat=4.3.0, -SeuratObject=4.1.3, -SingleCellExperiment=1.18.1, -SparseM=1.81, -SummarizedExperiment=1.26.1, -TSP=1.2-1, -UpSetR=1.4.0, -XVector=0.36.0, -abind=1.4-5, -anndata=0.7.5.3, -arm=1.13-1, -arrow=10.0.0, -askpass=1.1, -assertthat=0.2.1, -backports=1.4.1, -base64enc=0.1-3, -beachmat=2.12.0, -bit=4.0.5, -bit64=4.0.5, -bitops=1.0-7, -blob=1.2.3, -boot=1.3-28.1, -brew=1.0-7, -brio=1.1.3, -broom=1.0.1, -bslib=0.4.1, -ca=0.71.1, -caTools=1.18.2, -cachem=1.0.6, -callr=3.7.3, -car=3.1-1, -carData=3.0-5, -cellranger=1.1.0, -checkmate=2.1.0, -circlize=0.4.15, -cli=3.4.1, -clipr=0.8.0, -clue=0.3-63, -cluster=2.1.4, -coda=0.19-4, -codetools=0.2-18, -colorspace=2.0-3, -commonmark=1.8.1, -corrplot=0.92, -cowplot=1.1.1, -cpp11=0.4.3, -crayon=1.5.2, -credentials=1.3.2, -crosstalk=1.2.0, -curl=4.3.3, -data.table=1.14.6, -dbplyr=2.1.1, -deldir=1.0-6, -dendextend=1.16.0, -desc=1.4.2, -devtools=2.4.3, -dichromat=2.0-0.1, -diffobj=0.3.5, -digest=0.6.31, -doParallel=1.0.17, -dplyr=1.0.10, -dqrng=0.3.0, -dtplyr=1.2.2, -edgeR=3.38.4, -egg=0.4.5, -ellipsis=0.3.2, -emmeans=1.8.2, -estimability=1.4.1, -evaluate=0.19, -expm=0.999-6, -fansi=1.0.3, -farver=2.1.1, -fastmap=1.1.0, -feather=0.3.5, -figpatch=0.2, -fitdistrplus=1.1-8, -fontawesome=0.4.0, -forcats=0.5.2, -foreach=1.5.2, -foreign=0.8-83, -formatR=1.12, -formattable=0.2.1, -fs=1.5.2, -futile.logger=1.4.3, -futile.options=1.0.1, -future=1.29.0, -future.apply=1.10.0, -gargle=1.2.1, -gclus=1.3.2, -generics=0.1.3, -gert=1.6.0, -ggalluvial=0.12.3, -ggcorrplot=0.1.4, -ggplot2=3.4.0, -ggplotify=0.1.0, -ggpubr=0.5.0, -ggrepel=0.9.2, -ggridges=0.5.4, -ggsci=2.9, -ggsignif=0.6.4, -gh=1.3.0=0.1.1, -globals=0.16.2, -glue=1.6.2, -goftest=1.2-3, -googledrive=2.0.0, -googlesheets4=1.0.1, -gplots=3.1.3, -gprofiler2=0.2.1, -gridBase=0.4-7, -gridExtra=2.3, -gridGraphics=0.5-1, -gtable=0.3.1, -gtools=3.9.4, -haven=2.5.1, -heatmaply=1.4.0, -here=1.0.1, -highr=0.9, -hms=1.1.2, -htmlTable=2.4.1, -htmltools=0.5.4, -htmlwidgets=1.5.4, -httpuv=1.6.6, -httr=1.4.4, -ica=1.0-3, -ids=1.0.1, -igraph=1.3.5, -ini=0.3.1, -interp=1.1-3, -irlba=2.3.5.1, -isoband=0.2.6, -iterators=1.0.14, -jpeg=0.1-9, -jquerylib=0.1.4, -jsonlite=1.8.4, -knitr=1.41, -labeling=0.4.2, -lambda.r=1.2.4, -later=1.3.0, -lattice=0.20-45, -latticeExtra=0.6-30, -lazyeval=0.2.2, -leiden=0.4.3, -lifecycle=1.0.3, -limma=3.52.4, -listenv=0.8.0, -lme4=1.1-31, -lmtest=0.9-40, -locfit=1.5-9.6, -lubridate=1.8.0, -magick=2.7.3, -magrittr=2.0.3, -mapproj=1.2.9, -maps=3.4.1, -matrixStats=0.63.0, -memoise=2.0.1, -mgcv=1.8-41, -mime=0.12, -miniUI=0.1.1.1, -minqa=1.2.5, -modelr=0.1.9, -munsell=0.5.0, -mvtnorm=1.1-3, -naniar=0.6.1, -network=1.17.2, -nlme=3.1-160, -nloptr=2.0.3, -nnet=7.3-18, -norm=1.0-10.0, -numDeriv=2016.8-1.1, -openssl=2.0.5, -openxlsx=4.2.5.1, -pacman=0.5.1, -pals=1.7, -parallelly=1.32.1, -patchwork=1.1.2, -pbapply=1.6-0, -pbkrtest=0.5.1, -pheatmap=1.0.12, -pillar=1.8.1, -pkgbuild=1.3.1, -pkgconfig=2.0.3, -pkgload=1.3.2, -pkgmaker=0.32.2, -plotly=4.10.1, -plyr=1.8.8, -png=0.1-8, -polyclip=1.10-4, -polynom=1.4-1, -praise=1.0.0, -prettyunits=1.1.1, -processx=3.8.0, -progress=1.2.2, -progressr=0.12.0, -promises=1.2.0.1, -ps=1.7.2, -purrr=0.3.5, -qap=0.1-2, -quantreg=5.94, -rappdirs=0.3.3, -rcmdcheck=1.4.0, -readr=2.1.3, -readxl=1.4.1, -registry=0.5-1, -rematch=1.0.1, -rematch2=2.1.2, -remotes=2.4.2, -renv=0.15.5, -reprex=2.0.2, -reshape2=1.4.4, -reticulate=1.26, -rhdf5=2.40.0, -rhdf5filters=1.8.0, -rjson=0.2.21, -rlang=1.0.6, -rmarkdown=2.17, -rngtools=1.5.2, -roxygen2=7.1.2, -rpart=4.1.19, -rprojroot=2.0.3, -rstatix=0.7.1, -rstudioapi=0.13, -rversions=2.1.1, -rvest=1.0.3, -sass=0.4.4, -scCB2=1.6.0, -scales=1.2.1, -scattermore=0.8, -sctransform=0.3.5, -scuttle=1.6.3, -selectr=0.4-2, -seriation=1.4.0, -sessioninfo=1.2.2, -shape=1.4.6, -shiny=1.7.3, -sitmo=2.0.2, -sna=2.7, -snow=0.4-4, -sourcetools=0.1.7, -sp=1.5-1, -sparseMatrixStats=1.8.0, -spatstat.data=3.0-0, -spatstat.explore=3.0-5, -spatstat.geom=3.0-3, -spatstat.random=3.0-1, -spatstat.sparse=3.0-0, -spatstat.utils=3.0-1, -statnet.common=4.6.0, -stringi=1.7.8, -stringr=1.5.0, -survival=3.4-0, -svglite=2.1.0, -sys=3.4.1, -systemfonts=1.0.4, -tensor=1.5, -testthat=3.1.6, -tibble=3.1.8, -tidyr=1.2.1, -tidyselect=1.2.0, -tidyverse=1.3.2, -tinytex=0.42, -tzdb=0.3.0, -usethis=2.1.5, -utf8=1.2.2, -uuid=1.1-0, -uwot=0.1.14, -vctrs=0.5.1, -viridis=0.6.2, -viridisLite=0.4.1, -visdat=0.5.3, -vroom=1.6.0, -waldo=0.4.0, -webshot=0.5.4, -whisker=0.4, -withr=2.5.0, -xfun=0.35, -xml2=1.3.3, -xopen=1.0.0, -xtable=1.8-4, -yaml=2.3.6, -yulab.utils=0.0.5, -zip=2.2.0, -zlibbioc=1.42.0, -zoo=1.8-11

For manuscripts utilizing custom algorithms or software that are central to the research but not yet described in published literature, software must be made available to editors and reviewers. We strongly encourage code deposition in a community repository (e.g. GitHub). See the Nature Portfolio guidelines for submitting code & software for further information.

Data

Policy information about availability of data

All manuscripts must include a data availability statement. This statement should provide the following information, where applicable:

- Accession codes, unique identifiers, or web links for publicly available datasets
- A description of any restrictions on data availability
- For clinical datasets or third party data, please ensure that the statement adheres to our policy

Sequencing data generated in this study have been deposited in NCBI's Gene Expression Omnibus (GEO) and can be accessed through the GEO SuperSeries accession number GSE240020. Accession numbers for publicly available raw count data that have been re-analyzed for this study are listed here: (i) [Mouse] > Kernfeld et al. (2018) [GSE107910]; Bornstein et al. (2018) [GSE103967]; Dhalla et al. (2019) [https://www.ebi.ac.uk/biostudies/arrayexpress/studies/E-MTAB-8105#]; Baran-Gale et al. (2020) [https://bioconductor.org/packages/release/data/experiment/html/MouseThymusAgeing.html]; Wells et al. (2020)

[GSE137699]; Rota et al. (2021) [GSE162668]; Nusser et al. (2022) [GSE106856]; Michelson et al. (2022) [GSE194253]; Klein et al. (2023) [GSE215418]; Farley et al. (2023) [GSE232765]; Givony et al. (2023) [GSE236075]; Michelson et al. (2023) [GSE225661]; Horie et al. (2023) [GSE228198] and (ii) [Human] > Park et al. (2020) [https://zenodo.org/records/3711134]; Bautista et al. (2021) [GSE147520]; Ragazzini et al. (2023) [GSE220830, GSE220206, GSE220829]. The re-analyzed public datasets with added metadata can be accessed at 10.5281/zenodo.12516405.

Research involving human participants, their data, or biological material

Policy information about studies with human participants or human data. See also policy information about sex, gender (identity/presentation), and sexual orientation and race, ethnicity and racism.

Reporting on sex and gender	N/A
Reporting on race, ethnicity, or other socially relevant groupings	N/A
Population characteristics	N/A
Recruitment	N/A
Ethics oversight	N/A

Note that full information on the approval of the study protocol must also be provided in the manuscript.

Field-specific reporting

Please select the one below that is the best fit for your research. If you are not sure, read the appropriate sections before making your selection.

☒ Life sciences ☐ Behavioural & social sciences ☐ Ecological, evolutionary & environmental sciences

For a reference copy of the document with all sections, see nature.com/documents/nr-reporting-summary-flat.pdf

Life sciences study design

All studies must disclose on these points even when the disclosure is negative.

Sample size	Sample sizes were based on previously published work and preliminary studies, the effect size (difference between groups/standard deviation) for each study was estimated, and sample sizes were calculated using G*Power (v3.1.9.2) (Faul et al. 2009 Behavior Res Meth. 41:1149-1160) for an alpha=0.05 and beta=0.8.
Data exclusions	No samples were excluded from scRNA-seq, HALO or flow cytometry datasets.<br><br>scRNA-seq cell exclusions: Quality of the single cells was computationally assessed based on total counts, number of genes, mitochondrial and ribosomal fraction per cell, with low total counts, low number of genes (≤1000) and high mitochondrial content (≥0.2) as negative indicators of cell quality. Cells characterized by more than one negative indicator were considered as “bad” quality cells. Although cells were negatively sorted prior to sequencing for the CD45 marker, a small amount of CD45+ cells (expressing Ptprc), and also a few parathyroid cells (expressing Gcm2), were detected within our dataset. To remove bad quality cells and contaminants in an unbiased way, we assessed them in a cluster basis rather than individually. Leiden clusters with a “bad” quality profile and/or a high number of contaminating cells were removed. Finally, cells marked as doublets by scrublet were also filtered out.
Replication	In general variation within groups and between experiments was low, however, to take into account inter-experimental variation all experiments were performed at least twice and in no instance were experiments discarded due to conflicting findings. To account for intra-experimental variation, particularly for in vitro studies, several wells per conditions were assessed with primary sample material coming from at least two different mice. In vitro experiments were similarly independently repeated at least three times.
Randomization	No specific method of randomization was used. However, for all experiments, mouse cages were randomly allocated to each group after balancing of age and sex.
Blinding	Due to practical considerations in experimental design and the fact that aged mice are typically easy to distinguish from young mice, the performing investigator was not typically blinded to the group allocation during the experiments.

Reporting for specific materials, systems and methods

We require information from authors about some types of materials, experimental systems and methods used in many studies. Here, indicate whether each material, system or method listed is relevant to your study. If you are not sure if a list item applies to your research, read the appropriate section before selecting a response.

Materials & experimental systems

n/a	Involved in the study
☐	☒ Antibodies
☐	☒ Eukaryotic cell lines
☒	☐ Palaeontology and archaeology
☐	☒ Animals and other organisms
☒	☐ Clinical data
☒	☐ Dual use research of concern
☒	☐ Plants

Methods

n/a	Involved in the study
☒	☐ ChIP-seq
☐	☒ Flow cytometry
☒	☐ MRI-based neuroimaging

Antibodies

Antibodies used	The antibodies used were rabbit polyclonal anti-mouse Keratin 14 (905301, Biolegend), rat anti-mouse Keratin 8 (Troma-I; Developmental Studies Hybridoma Bank, Iowa City), and rabbit anti-mouse NKp46 (ab233558, Abcam). Secondary antibodies used were Opal 690 anti-rabbit (Akoya), Opal 520 anti-rat (Immpress), and Opal 570 anti-NKp46 (Akoya).<br>For flow cytometry and cell sorting, surface antibodies against CD45 (565967, BD Biosciences; 103154 BioLegend), CD31 (102434, Biolegend), CD140a (135907, Biolegend), MHC-II (107620, Biolegend), EpCAM (46-5791-82, BD Biosciences), Ly-51 (740882, BD Biosciences), UEA-1 (ZC0426, Vector Labs), DLCK-1 (NBP1-77127F, Novus Biologicals), Ly6D (138605, Biolegend), CD104 (123615, Biolegend), CD140a (135921, Biolegend), CD31 (102427, Biolegend), PDPN (127425, Biolegend), CD8a (100714, Biolegend), CD4 (565709, BD Biosciences), TCR-[illegible] (109239, Biolegend), CD3e (100232, Biolegend), CD25 (102030, Biolegend), CD44 (612799, BD Biosciences), NK1.1 (108753, Biolegend), CD49b (561067, BD Biosciences), c-Kit (105811, Biolegend), TCR-γ[illegible] (118107, Biolegend), CD1d PBS-57 Tetramer (NIH tetramer Core), CD11c (35-0114, Tonbo), CD11c (612796, BD Biosciences), CD11b (741722, BD Biosciences), XCR1 (148225, Biolegend), B220 (103232, Biolegend), CD127 (50-1271, Tonbo), Sca-1 (122527, Biolegend), CD135 (135305, Biolegend), CD150 (46-1502-82, eBioscience), CD48 (103427, Biolegend), NKG2D (562800, BD Biosciences), CD49a (741976, BD Biosciences), KLRG1 (138425, Biolegend), CCR-6 (129814, Biolegend), IL-23R (150907, Biolegend), ST2 (566310, BD Biosciences), H2-kb (116525, Biolegend), IL-18R (25-5183-82, Thermo Fisher), IL-18R (25-5183-82, Thermofisher) were used. Following fixation and permeabilization (554714, BD Biosciences), cells were stained with the following antibodies: Perforin (154315, Biolegend), Granzyme B (MHGB04, Thermofisher). Annexin V and 7-AAD staining (640920, BioLegend) was performed in Annexin V binding buffer (422201, BioLegend). A detailed table outlining specific vendors, fluorochromes, catalog numbers, lot numbers and dilutions has been included in a separate data table.
Validation	Isotype, fluorescent-minus one, and genetic knockout (when appropriate) controls were used to set gates.

Eukaryotic cell lines

Policy information about cell lines and Sex and Gender in Research

Cell line source(s)	OP9-DLL1 and RMA(S)
Authentication	N/A
Mycoplasma contamination	N/A
Commonly misidentified lines<br>(See ICLAC register)	N/A

Animals and other research organisms

Policy information about studies involving animals; ARRIVE guidelines recommended for reporting animal research, and Sex and Gender in Research

Laboratory animals	Inbred male and female C57Bl/6J (000664) and B6 CD45.1 (002014) mice were obtained from Jackson Laboratories (Bar Harbor, USA). Il1r1-/- (003245), Il18-/- (004130), Il18r1-/- (004131), Casp1[illegible]10 (032662), CD1d-/- (008881), Ifngr-/- (003288), Prf1-/- (002407), Rag2-eGFP (005688) and GREAT (“interferon-gamma reporter with endogenous polyA transcript”) mice were obtained from Jackson Laboratories and bred in house. Gzmb-/- mice were obtained from Geoffrey Hill (Fred Hutchinson Cancer Center) and bred in house. Il18 flox mice (Il18fl/fl) were obtained from R. Nowarski (Harvard Medical School) and R. Flavell (Yale School of Medicine) and crossed in house to Zbtb46 Cre+ obtained from Jackson Laboratories (032662) to generate Il18fl/fl Zbtb Cre+ (Il18[illegible]DC) mice. Il18r1fl/fl mice were obtained from G. Trinchieri (NCI) and crossed to Foxn1 Cre+ mice obtained from Jackson Laboratories (018448) and Ncr1 Cre+ obtained from K. Barry (Fred Hutch Cancer Center) to generate Il18r1fl/fl Foxn1 Cre+ (Il18r1[illegible]TEC) and Il18r1fl/fl Ncr1 Cre+ (Il18r1[illegible]NK), respectively. Ifngrfl/fl (025394) and Foxn1 Cre+ (018448) mice were obtained from Jackson Laboratories and crossed to generate Ifngrfl/fl Foxn1 Cre+ (Ifngr[illegible]TEC) mice. All experimental mice were between 6-10 weeks old. Animal rooms were kept in a constant temperature of 72 degrees (+/- 3 degrees) Fahrenheit with humidity set between 30%-70% as recommended in the "Guide for the Care and Use of Lab Animals". Light cycles were set for 12 hours on and 12 hours off.
Wild animals	No wild animals were used in this study.
Reporting on sex	All mouse ages and sexes are reported in figure legends

Field-collected samples	No field collected samples were used in this study.
Ethics oversight	All studies were performed under approved Institutional Animal Care and Use Committee (or equivalent) protocols at Fred Hutchinson Cancer Center and Memorial Sloan Kettering Cancer Center.

Note that full information on the approval of the study protocol must also be provided in the manuscript.

Flow Cytometry

Plots

Confirm that:

☒ The axis labels state the marker and fluorochrome used (e.g. CD4-FITC).

☒ The axis scales are clearly visible. Include numbers along axes only for bottom left plot of group (a 'group' is an analysis of identical markers).

☒ All plots are contour plots with outliers or pseudocolor plots.

☒ A numerical value for number of cells or percentage (with statistics) is provided.

Methodology

Sample preparation	Thymus was enzymatically digested following and adapted protocol. Briefly, thymi were mechanically dissociated into 1-2 mm pieces. Tissue pieces were incubated with a digestion buffer (either, RPMI with 10% FCS, 62.5 um/mL liberase TM, 0.4 mg/ml DNase I; or RPMI with 25mM HEPES, 20µg/µL DNAse1, and 1mg/mL Collagenase/Dispase). Between incubation steps, supernatant containing dissociated cells were transferred to tubes equipped with 100 um filter. Cells were pelleted by centrifugation at 400g for 5 min. All steps were performed at 4ºC unless indicated. For sequencing experiments, cell pellets were incubated with anti-mouse CD45 microbeads and CD45+ cells were depleted from cell suspension using magnetic-associated cell sorting (MACS) on LS columns according to manufacturer's protocol. Following red blood cell lysis using ACK buffer, the CD45-depleted cell fraction was incubated with an antibody cocktail for 15 min at 4 C and cells of interest were purified by fluorescent-associated cell sorting (FACS) on a BD Biosciences Aria II using a 100 micro meter nozzle. Cells were sorted into tubes containing RPMI supplemented with 2% BSA. FACS-purified cells were spun down at 400g for 5 min and resuspended in PBS supplemented with 0.04 % BSA for generation of single-cell suspensions. Isolation of thymus NK and CD45- cells for ex vivo cytotoxicity assays is detailed in figure legends.
Instrument	Cells sorting was performed on a BD Biosciences Aria II or S6 using a 100 um or 70um nozzle. Flow cytometry analysis was performed on BD Symphony A6.
Software	Flow cytometry was analyzed using FlowJo (Treestar Software).
Cell population abundance	For sort experiments, cell purity was typically >99% as checked using a post-sort purity check by flow cytometry.
Gating strategy	All gating have been outlined in the manuscript.

☒ Tick this box to confirm that a figure exemplifying the gating strategy is provided in the Supplementary Information.
